# Gut microbiome and urinary metabolomic alterations in Chinese infants with functional constipation and yin-deficiency constitution

**DOI:** 10.3389/fmicb.2026.1851053

**Published:** 2026-08-14

**Authors:** Haihong Zhao, Xiuxia Lu, Yanbin Ye, Hui Ye, Weiwei Li, Ziyu Long, Xuezhi Zhang, Jieling Wu, Ji Wang

**Affiliations:** 1 Traditional Chinese Medicine and Integrative Medicine Department, Peking University First Hospital, Peking University, Beijing, China; 2 Department of Children’s Health Care, Guangdong Women and Children Hospital, Guangzhou, China; 3 Faculty of Chinese Medicine Science, Traditional Chinese Medicine School, Guangxi University of Chinese Medicine, Nanning, China; 4 National Institute of TCM Constitution and Preventive Treatment of Disease, Beijing University of Chinese Medicine, Beijing, China

**Keywords:** functional constipation, infants, metabolome, microbiota, yin-deficiency constitution

## Abstract

**Background:**

Functional constipation (FC) is common in early childhood and is closely associated with gut microbiota imbalance. According to Traditional Chinese Medicine (TCM) constitution theory, infants can be categorized into different constitution types, among which the yin-deficiency constitution is particularly linked to a higher risk of FC. However, data specifically addressing this association in infants remain limited.

**Methods:**

Infants under 2 years of age were assessed for yin-deficiency and balanced constitutions using a TCM Constitution Questionnaire, and FC was diagnosed according to the Rome IV criteria. The study enrolled 31 infants with yin-deficiency constitution and FC (YINDC) and 31 infants with balanced constitution and without FC (BC, controls). Evaluations included Bristol Stool Scale scores, constipation symptoms, fecal microbiota profiles, and urinary metabolite characteristics.

**Results:**

The YINDC group exhibited significantly lower Bristol Stool Scale scores and more severe FC symptoms than the BC group. Marked differences in gut microbial composition and metabolite profiles were observed between the two groups, with main alterations identified in the sphingolipid signaling and metabolism pathways.

**Conclusion:**

This study compares gut microbial and urinary metabolomic profiles between infants with the combined yin-deficiency constitution/FC phenotype and balanced non-constipated controls. We identified preliminary microbiota and metabolomic differences between the two groups, involving sphingolipid-related pathways. However, these differences cannot be specifically attributed to yin-deficiency constitution, FC, or their interaction. Our findings provide a preliminary basis for future investigation of the gut microbiome-metabolome axis in TCM-integrated FC management, but require validation in independent cohorts.

## 1 Introduction

In pediatric populations, over 90% of constipation cases stem from functional factors (Loening-Baucke, 1993; Loening-Baucke, 2005). Functional constipation (FC) is characterized by persistent difficulties in bowel movements, incomplete defecation, or reduced bowel movement frequency without organic causes, resulting in significant patient distress and increased healthcare costs (Vriesman et al., 2019). TCM constitution theory categorizes individuals into nine types, each with distinct etiologies and clinical characteristics. Balanced constitution refers to a health state in which illness rarely occurs; in contrast, yin-deficiency constitution, one of the most common constitution types among infants, may arise from various factors including parental constitution types, inappropriate maternal lifestyle during pregnancy (such as excessive consumption of fried foods and emotional stress), specific environmental conditions, and the infant’s disease history (Li and Wang, 2012; Zhao et al., 2022). The primary characteristic of this constitution is a deficiency of body fluids, resulting in internal heat (Zhao et al., 2023).

Infants with yin-deficiency constitution exhibit insufficient body fluids, which reduces stool moisture content, leading to dry, hard, and difficult-to-pass feces. Furthermore, dehydration diminishes intestinal lubrication, resulting in less frequent and weaker peristaltic contractions that further aggravate constipation. Multiple questionnaire-based studies of infants with FC have reported that those with yin-deficiency constitution account for 24.4–30.41% of cases (Chen, 2024; Wang, 2023; Yang, 2023). In addition, yin-deficiency constitution has been identified as one of the two predisposing constitution types for FC in infants (Yang, 2023). Given these findings, investigating the mechanisms linking yin-deficiency constitution and FC in infants is essential and may hold considerable clinical relevance for developing targeted interventions.

Although our previous studies have individually analyzed gut microbiota in constipated infants and in infants with yin-deficiency constitution (Zhao et al., 2023; Zhao et al., 2025), no specific analysis has been conducted for infants with both conditions (i.e., yin-deficiency constitution combined with FC, hereafter referred to as YINDC). Considering the notable proportion of YINDC infants within the FC population, this study collected fecal and urine samples from YINDC infants aged 0–2 years for 16S rRNA gene sequencing and UPLC-Q-TOF/MS analysis. The findings may provide a basis for developing targeted strategies to manage constipation in this subgroup. Notably, this study employs a cross-sectional case-control design. Because yin-deficiency constitution and constipation are treated as a single entity, the independent effects of constitution and constipation cannot be distinguished.

## 2 Materials and methods

### 2.1 Participants

From November 2022 to April 2023, we enrolled infants aged 0–2 years through online advertisements and physical postings. Participants’ constitution types were identified using Academician Wang Qi’s classification instrument, as detailed in Supplementary File 1. This questionnaire consists of 43 items corresponding to the nine constitution types and has demonstrated excellent reliability and validity (Li et al., 2019; Li, 2024; Lu et al., 2022). The questionnaire was completed by the infant’s primary caregiver, and participants were assigned to a specific constitution type when their transformation scores met the predefined thresholds.

The Rome IV criteria were applied to establish the diagnosis of FC. Diagnosis required at least two of the following symptoms to be present for a minimum of 1 month: (1) two or fewer defecations per week; (2) a history of excessive stool retention; (3) a history of painful or hard bowel movements; (4) a history of large-diameter stools; (5) the presence of a large fecal mass in the rectum. For non-toilet-trained infants, only the five Rome IV criteria that do not depend on toileting behavior were used (Yang, 2023).

Infants identified with yin-deficiency constitution and FC (YINDC group) and those with balanced constitution without FC (BC group, controls) were included in this study. Exclusion criteria included recent use (within 3 months) of antibiotics or probiotics, prior gastrointestinal surgery, and a diagnosis of neuropsychiatric or other severe systemic disorders.

### 2.2 Collection and processing of fecal samples

Stool samples were collected by guardians. Two scoops of urine-free feces were placed in a sterile cup and transported to the hospital on an ice pack within 1 h. Each sample was then immediately flash-frozen and stored at -80°C. Genomic DNA was extracted using the CTAB/SDS method, and its quality and concentration were evaluated by agarose gel electrophoresis. PCR amplification targeted the V3-V4 hypervariable region of the 16S rRNA gene. The resulting amplicons were purified to generate sequencing libraries, which were subsequently sequenced on the Illumina MiSeq platform for high-throughput data generation (Lv et al., 2023).

### 2.3 Gut microbiota analysis

Microbiome bioinformatics was performed with QIIME2 2022.11 (Bolyen et al., 2019). Briefly, raw sequence data were demultiplexed using the demux plugin, followed by primers cutting with cutadapt plugin (Marcel, 2011). Sequences were then quality filtered, denoised, merged and chimera removed using the DADA2 plugin (Callahan et al., 2016). Non-singleton amplicon sequence variants (ASVs) were aligned with MAFFT (Katoh et al., 2002) and used to construct a phylogeny with FastTree 2 (Price et al., 2009). α and β diversity of the microbial communities were estimated. To identify differentially abundant taxa, LEfSe analysis was performed with an LDA score threshold > 2.0 (Kanehisa and Goto, 2000; Kanehisa et al., 2025; Kanehisa, 2019; Langille et al., 2013).

### 2.4 Collection and processing of urine samples

Guardians collected the middle portion of morning urine at home and transferred it to the hospital on an ice pack within 1 h. After centrifugation, the supernatant was rapidly frozen and preserved at -80°C. For metabolite extraction, samples were shipped on dry ice to Shanghai Applied Protein Technology Co., Ltd. (Wen et al., 2021). The extraction procedure involved thawing, mixing with a methanol/acetonitrile solvent, sonication, and centrifugation. The resultant supernatant was dried, then re-dissolved in an acetonitrile/water solution, vortexed, and centrifuged again. Finally, ultra-high-performance liquid chromatography coupled with quadrupole time-of-flight mass spectrometry (UHPLC-Q-TOF MS, TOF 5600 + system) was used for analysis, ensuring accurate and reliable metabolomic profiling.

### 2.5 Metabolomics analysis

Raw data in mzXML format were processed using the XCMS 3.10.1 package (Scripps, La Jolla, CA, United States), which performed peak alignment, retention time correction, and peak area extraction (Chambers et al., 2012; Jia et al., 2018). Subsequent steps included metabolite identification, preprocessing, and quality evaluation. For statistical analysis, fold change was used for univariate analysis. Multivariate analysis via orthogonal partial least squares discriminant analysis (OPLS-DA) identified global metabolic differences between the YINDC and BC groups. Variable importance in projection (VIP) values helped determine characteristic metabolites in the two groups. Pathway analysis was conducted using MetaboAnalyst web software, and pathway enrichment significance was assessed with Fisher’s exact test. Missing metabolomics data were imputed using the KNN method. It should be noted that urinary metabolite intensities were analyzed as raw peak area intensities without normalization to creatinine, specific gravity, osmolality, total ion current, or probabilistic quotient normalization.

### 2.6 Statistical analysis

Spearman correlation analysis, visualized by heatmaps, was performed using R and Cytoscape to determine relationships between differential flora and metabolites. For microbiome studies, box plots generated via R scripts illustrated α diversity differences between the YINDC and BC groups. Statistical significance was validated using Kruskal-Wallis rank-sum tests with Dunn’s *post-hoc* tests, while PERMANOVA was used for inter-group differential analysis. For metabolomics, *t*-tests with false discovery rate (FDR) correction were applied. All statistical analyses were performed using SPSS and GraphPad Prism software, with statistical significance set at *P* < 0.05.

## 3 Results

### 3.1 Constipation status of participants

Following questionnaire screening, constitution differentiation, and confirmation of FC status, 31 BC infants and 31 YINDC infants were enrolled in the study. No significant differences were observed between the two groups in terms of age, sex, delivery mode, or feeding patterns (Supplementary File 2). Stool consistency, assessed by the Bristol Stool Scale (BSS), differed markedly between the groups. The BC group predominantly passed soft blobs, whereas the YINDC group mainly produced sausage-shaped stools with cracks—a pattern that is both age-related and closely associated with the clinical presentation of FC. BSS scores were significantly lower in the YINDC group than in the BC group (*P* < 0.001), indicating more pronounced constipation in YINDC infants (Table 1).

**TABLE 1:** Number of participants with BSS term in two groups.

BSS term	YINDC group	BC group	*P*-value
Separate hard lumps, nuts-like	7 (22.58%)	0	−
Sausage-shaped but lumpy	8 (25.81%)	0	−
Sausage-shaped with cracks	13 (41.94%)	1 (3.23%)	−
Smooth snake	2 (6.45%)	10 (32.26%)	−
Soft blobs	1 (3.23%)	13 (41.94%)	−
Fluffy pieces	0	6 (19.35%)	−
Watery	0	1 (3.23%)	−
Total number	31 (100%)	31 (100%)	< 0.001

In addition to BSS scoring, we assessed the prevalence of FC symptom. As shown in Table 1, the YINDC group had significantly higher proportions of symptoms including defecations ≤ 2 times/week (*P* < 0.001), painful/hard bowel movements (*P* < 0.001), and lexcessive stool retention (*P* < 0.001) compared with the BC group. However, no significant differences were observed for large-diameter stools (*P* = 0.147) or rectal fecal mass (*P* = 0.238) (Table 2).

**TABLE 2:** Prevalence of FC symptom in two groups.

FC symptom	YINDC group	BC group	*P*-value
Two or fewer defecations per week	17 (54.8%)	1 (3.2%)	<0.001
History of excessive stool retention	22 (71.0%)	0	<0.001
History of painful or hard bowel movements	21 (67.7%)	1 (3.2%)	<0.001
History of large-diameter stools	7 (22.6%)	2 (6.5%)	0.147
Presence of a large fecal mass in the rectum	3 (9.7%)	0	0.238

Because a single participant could present with more than one symptom simultaneously, the percentages for each symptom were calculated independently using the total number of participants in each group as the denominator. Therefore, the sum of percentages across symptoms may exceed 100%.

### 3.2 Gut flora diversity was altered in YINDC infants

**FIGURE 1:**
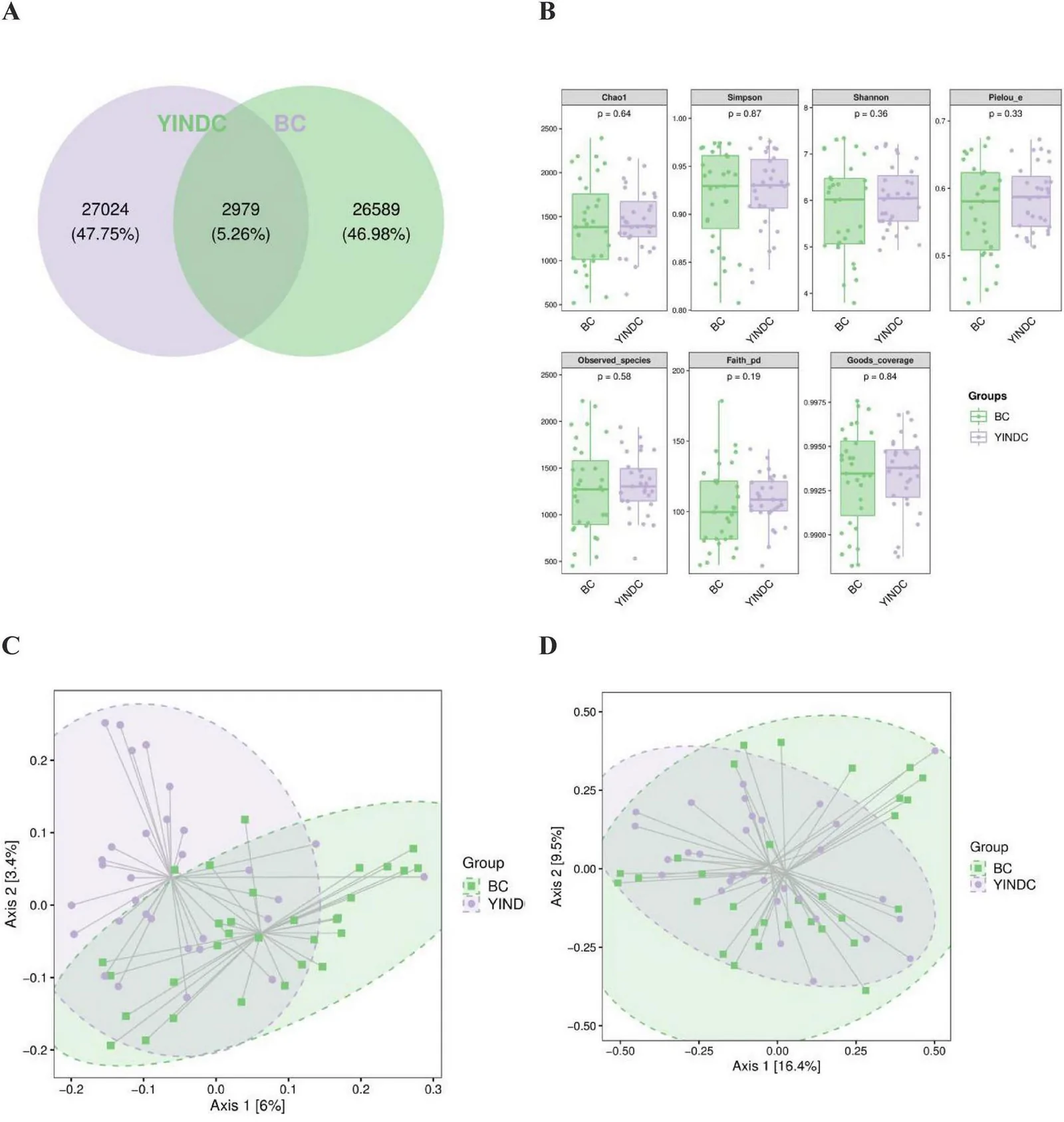
Altered gut microbial diversity in YINDC infants. **(A)** Venn diagram of ASVs. **(B)**α diversity consisting of many indicators. **(C)** β diversity based on unweighted unifrac. **(D)** β diversity based on weighted unifrac.

Gut microbial diversity was assessed via 16S rRNA gene sequencing. The YINDC and BC groups yielded 27,024 and 26,589 unique ASVs, respectively, with 2,979 shared between them (Figure 1A). The average sequencing depth was 109,379 reads per sample and the sequencing depth plots was shown in Supplementary File 3. Number of reads retained per sample was shown in Supplementary File 4. α diversity analysis revealed no statistically significant differences between the two groups for any metric (*P* > 0.05), indicating similar within-community bacterial diversity (Figure 1B).

To explore compositional differences, we conducted β diversity analysis. PCoA based on unweighted and weighted UniFrac distances revealed distinct clustering patterns separating the YINDC and BC groups, indicating significant disparities in bacterial composition between the two groups (Figures 1C,D). PERMANOVA based on unweighted and weighted UniFrac distances (999 permutations) showed significant separation between groups (*R*^2^ = 0.0237, *P* = 0.001 for unweighted; *P* = 0.021 for weighted). The dispersion test yielded *P* = 0.035, indicating a significant difference in within-group variability between the two groups.

### 3.3 Gut flora composition was altered in YINDC infants

**FIGURE 2:**
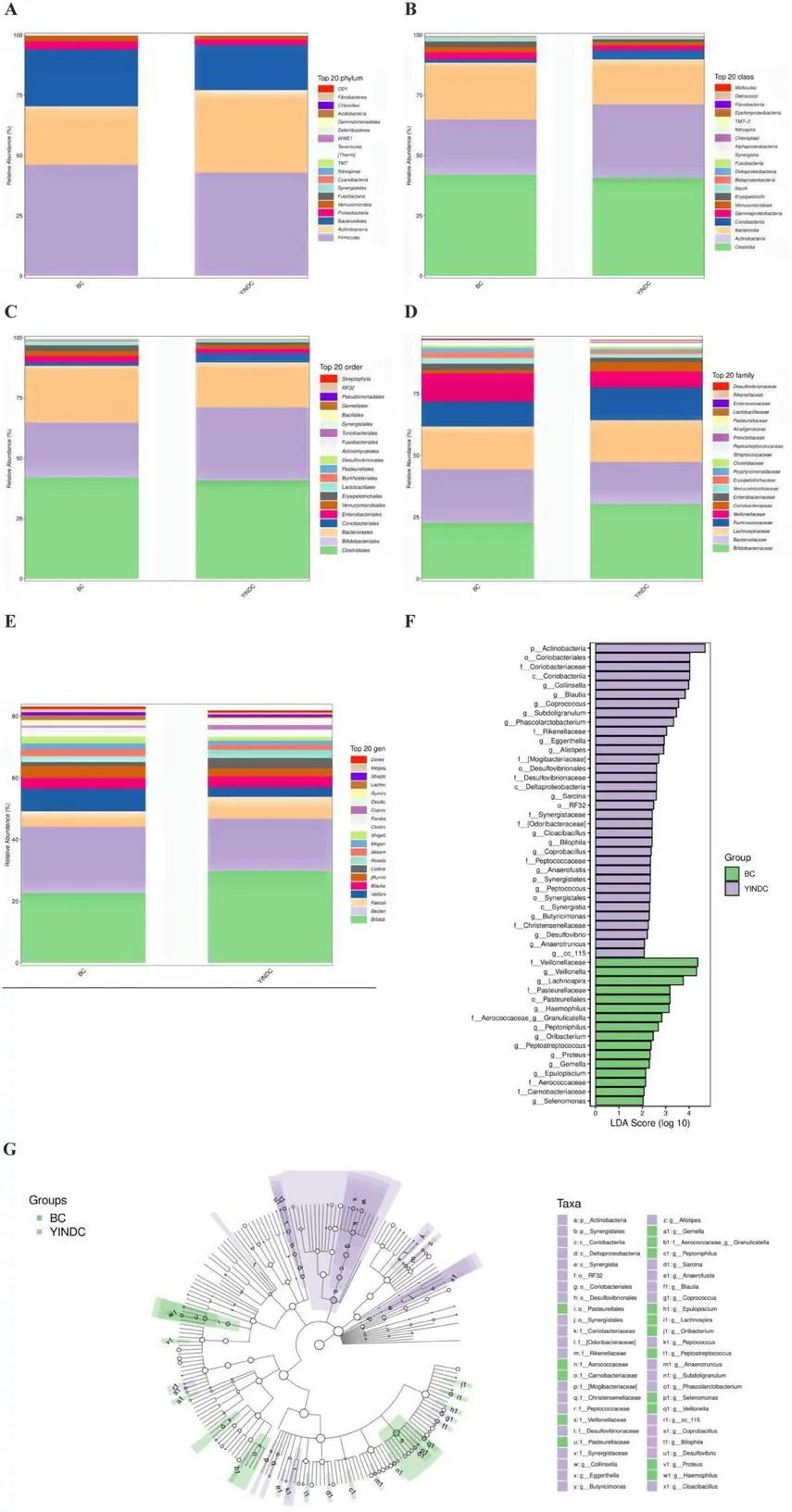
Altered gut microbial composition in YINDC infants. **(A–E)** Relative abundance of the top 20 taxa at the phylum, class, order, family, and genus levels, respectively. **(F)** LEfSe analysis identifying gut microbial features with an LDA score (log10) > 2. Bar length indicates the effect size (LDA score). **(G)** Cladogram illustrating the phylogenetic distribution of differentially abundant taxa across all taxonomic levels.

Microbial composition was examined at five taxonomic levels, with the top 20 taxa at each level in Figures 2A–E. LEfSe analysis, visualized via a cladogram (Figure 2G), enabled simultaneous comparison across all taxonomic levels and facilitated the identification of candidate taxa (Figure 2F). In total, 50 characteristic taxa were identified, including 34 enriched in the YINDC group and 16 in the BC group. Within the YINDC group, characteristic phyla comprised Actinobacteria and Synergistetes, while characteristic genera included *Collinsella*, *Blautia*, *Coprococcus*, *Subdoligranulum*, *Phascolarctobacterium*, *Eggerthella*, *Alistipes*, *Sarcina*, *Cloacibacillus*, *Bilophila*, *Coprobacillus*, *Anaerofustis*, *Peptococcus*, *Butyricimonas*, *Desulfovibrio*, and *Anaerotruncu*. In the BC group, the characteristic genera included *Veillonella*, *Lachnospira*, *Haemophilus*, *Granulicatella*, *Peptoniphilus*, *Oribacterium*, *Peptostreptococcus*, *Proteus*, *Gemella*, *Epulopiscium*, and *Selenomonas*.

### 3.4 Urinary metabolomic profiles were altered in YINDC infants

Urine, a terminal metabolic product, contains diverse metabolites that reflect the body’s biochemical status. Its non-invasive and non-infectious nature makes it particularly suitable for metabolic profiling in infants. For untargeted metabolomic analysis, we collected urine samples from 31 BC infants and 30 YINDC infants.

**FIGURE 3:**
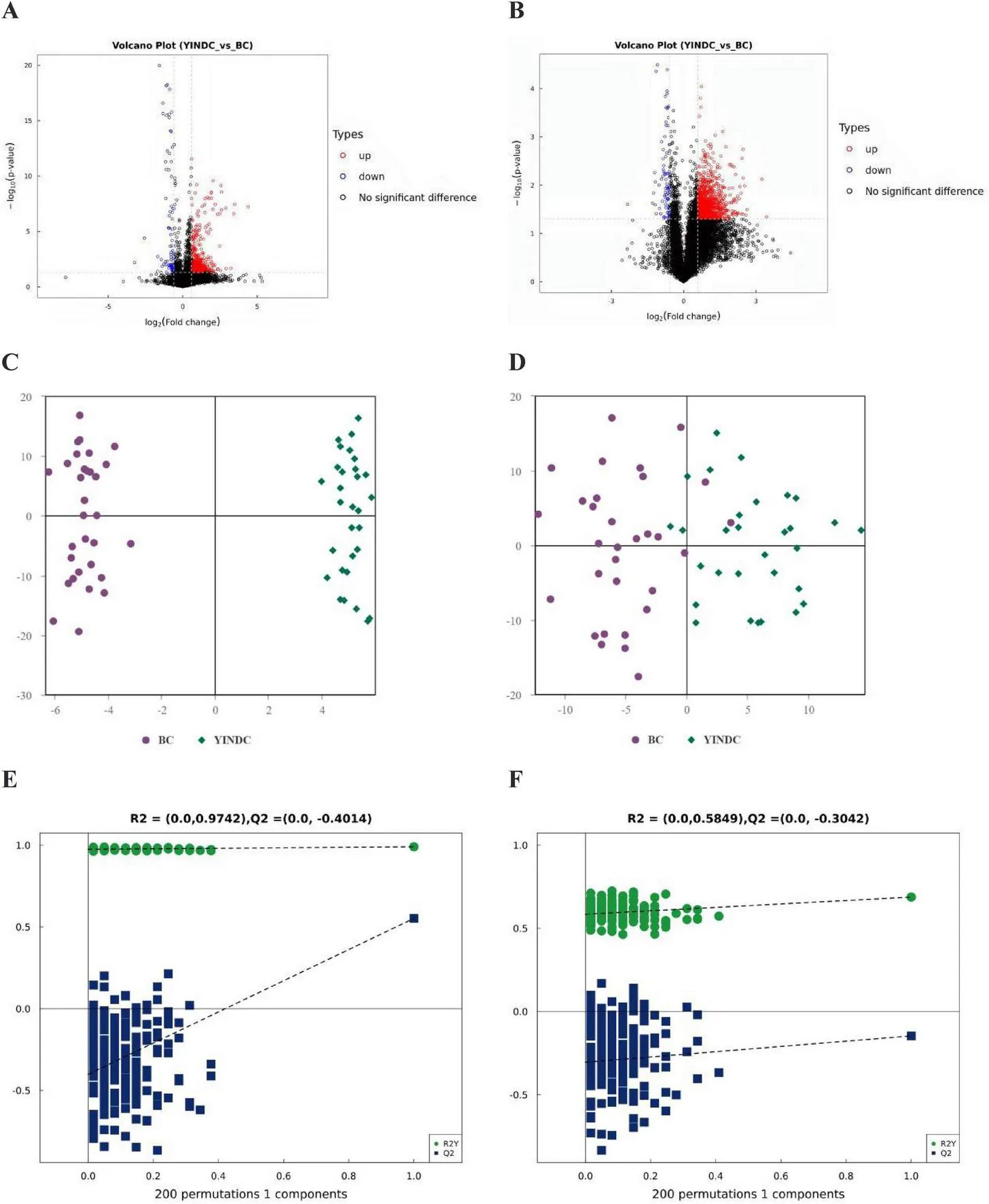
Altered urinary metabolome in YINDC infants. **(A)** Volcano plots of positive ion mode. **(B)** Volcano plots of negative ion mode. Rose represented differential metabolites with Fold change > 1.5, suggesting up-regulation of their expression in the YINDC group. Blue represented differential metabolites with Fold change < 0.67, suggesting down-regulation of their expression in the BC group. Black represented metabolites with no statistically significant difference. **(C)** OPLS-DA score graph for positive ion mode. **(D)** OPLS-DA score graph for negative ion mode. **(E)** Permutation test of OPLS-DA for positive ion mode. **(F)** Permutation test of OPLS-DA for negative ion mode.

Total ion flow plots of quality control (QC) sample showed substantial overlap, indicating minimal machine-related variation throughout the experiment (Supplementary File 5). Univariate analysis of all metabolites was performed, and the results were visualized using volcano plots (Figures 3a,b). OPLS-DA revealed clear separation between the YINDC and BC samples (Figures 3C–F), demonstrating that the model effectively distinguished the two groups. These results indicate that YINDC infants have significantly altered metabolite profiles compared to BC infants.

### 3.5 Differential metabolites were identified between groups

VIP values derived from OPLS-DA enabled the quantification and analysis of between-group differences in metabolite expression, facilitating the identification of significantly altered metabolites. Following the application of strict threshold (VIP > 1.0, *P* < 0.05 and *P* < 0.05 after FDR correction) and manual review, we identified 15 differential metabolites (Supplementary File 6). The MS/MS information were provided in Supplementary File 7.

### 3.6 Metabolic pathway was identified in YINDC infants

**FIGURE 4:**
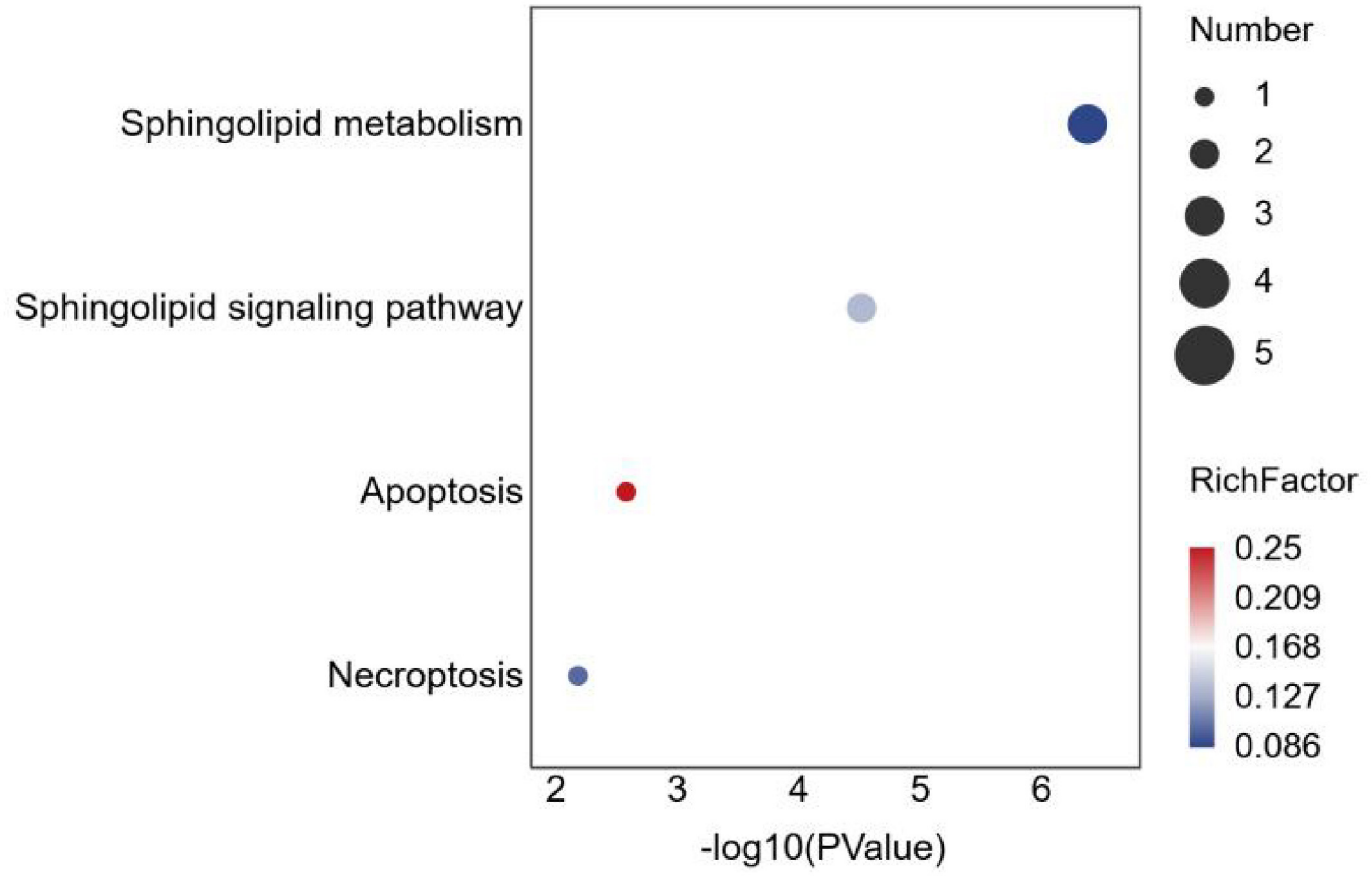
Differential abundance score plot illustrating the enriched metabolic pathways.

To explore potential metabolic pathways associated with YINDC, we mapped the differential metabolites to relevant physiological pathways. Differential abundance score analysis suggested alterations in sphingolipid signaling, sphingolipid metabolism, apoptosis, and necroptosis in the YINDC group (Figure 4). The specific information can be found in Table 3.

**TABLE 3:** Metabolic pathway information.

Metabolic pathway	Metabolite	*P*-value	FDR-corrected *P*-value	Rich factor	Pathway impact value
Sphingolipid metabolism pathway	Phytosphingosine, pro-trp, sphingosine	0.0000	0.0000	0.0857	0.09
Sphingolipid signaling pathway	Pro-trp, sphingosine	0.0000	0.0001	0.1333	0.13
Apoptosis pathway	Sphingosine	0.0026	0.0044	0.2500	0.25
Necroptosis pathway	Sphingosine	0.0066	0.0082	0.1000	0.1

### 3.7 Correlation analysis between differential microbial taxa and metabolites

**FIGURE 5:**
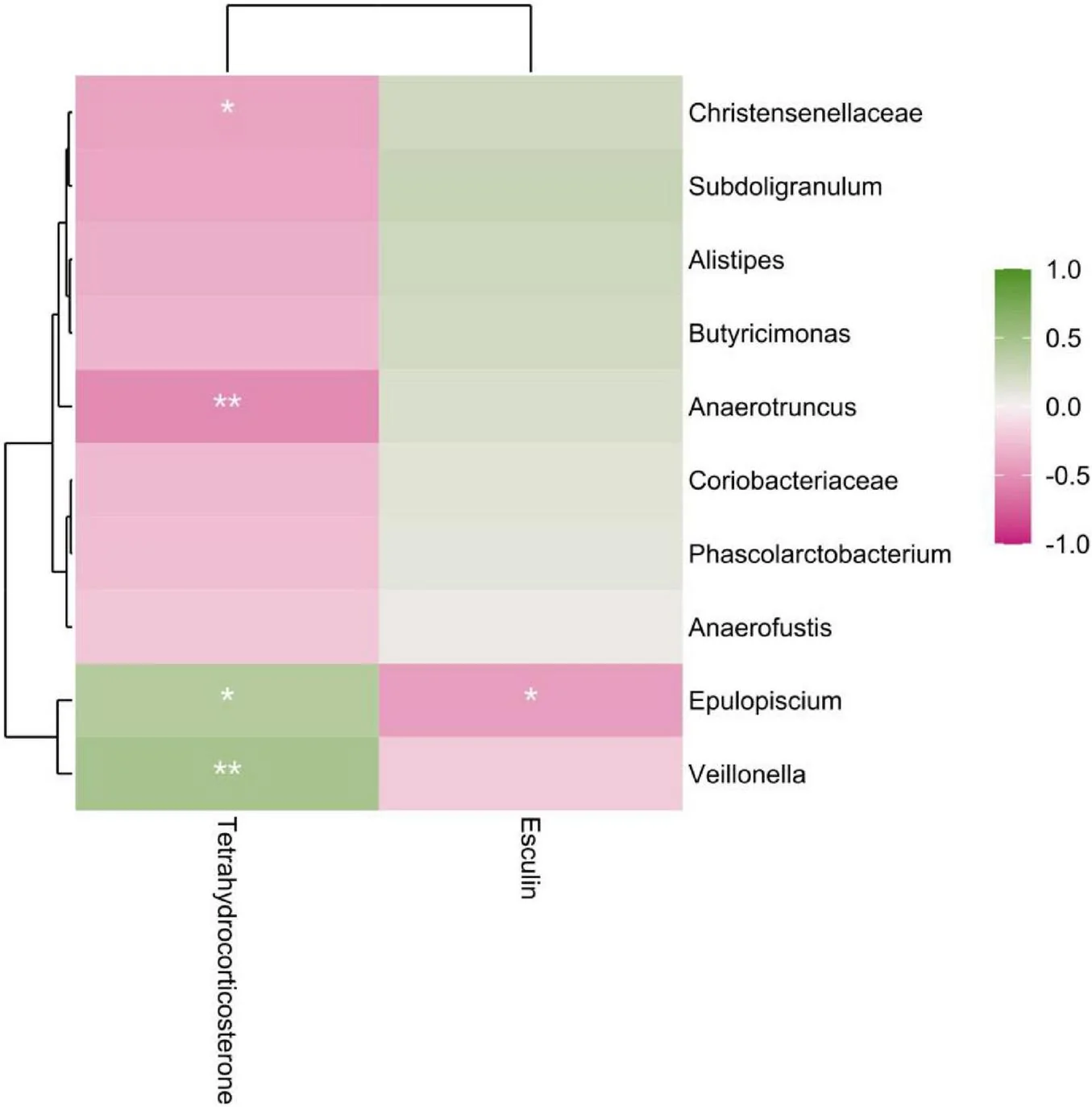
Correlation analysis between differential flora and metabolites. *FDR-adjusted *P*-value < 0.05, **FDR-adjusted *P* < 0.01.

To explore associations between gut microbiota and metabolic changes, we performed Spearman correlation analysis. In summary, 53 significant correlations after FDR correction were identified (FDR-adjusted *P* < 0.05), of which 11 pairs showed strong correlations (| *r*| > 0.5). The correlation heatmap following FDR correction was shown in Figure 5. Specifically, tetrahydrocorticosterone exhibited strong positive correlations with *Anaerotruncus*, and negative correlations *Veillonella.* These results suggest that specific gut microbes may be linked to metabolite levels.

## 4 Discussion

While we have previously reported microbiota and metabolomic profiles in FC and in damp-heat constitution, the current study focuses specifically on the YINDC phenotype, which has not been previously described. This research examined the gut microbiome and urinary metabolome of infants aged 0–2 years diagnosed with YINDC. Compared to healthy controls, the YINDC group exhibited marked gut dysbiosis and altered metabolite profiles, offering new perspectives on FC pathophysiology through the lens of TCM constitution theory.

Accumulating evidence underscores dysbiosis of the intestinal flora as a pivotal etiological factor in pediatric FC. Recent metagenomic studies have further illuminated the central role of bacterial composition and metabolic potential in the pathophysiology and progression of FC (de Meij et al., 2016; Mancabelli et al., 2017). The gut microbiota undergoes dynamic assembly and stabilization during the first 2 years of life, a critical window that coincides with rapid infant development (Robertson et al., 2019). In TCM, infants are considered to possess an excess of Yang Qi, which is prone to transforming into heat and depleting body fluids, predisposing them to a yin-deficiency state [Ye Tianshi, *Essentials of Pediatrics*]. This constitutional predisposition aligns with our previous epidemiological findings (Zhao et al., 2022), and forms the rationale for focusing on this vulnerable population. Our comparative analysis identified significant alterations at both the microbial and metabolic levels in YINDC infants.

A notable observation was the marked elevation of Synergistetes at the phylum level in the YINDC group. Although not previously linked to yin-deficiency constitution (Zhao et al., 2023), the elevated abundance of this often opportunistic phylum has been specifically associated with constipation in other contexts, such as antipsychotic-induced gastrointestinal hypomotility (Xu et al., 2022). This finding suggests that the rise in Synergistetes may be more directly related to the FC phenotype rather than the underlying constitution alone. Additionally, we observed a distinct microbial signature in the YINDC group, marked by elevated abundances of *Collinsella* and *Phascolarctobacterium*, coupled with decreased levels of *Veillonella*, *Selenomonas*, and *Haemophilus*.

The enrichment of *Collinsella*, a genus linked to low dietary fiber intake (Gomez-Arango et al., 2018), and its positive correlation with altered stool consistency (Li et al., 2018), underscores a potential diet-microbiota interaction. Similarly, the increase in *Phascolarctobacterium*, previously reported in constipated cohorts, reinforces its association with dysmotility (He et al., 2023; Jalanka et al., 2019). Conversely, the depletion of beneficial taxa such as *Veillonella*, *Selenomonas*, and *Haemophilus* collectively paints a picture of a gut ecosystem shifted away from a motility-promoting state. Specifically, *Veillonella* predominates in the healthy small intestinal ecosystem, which is consistent with its high abundance observed in the BC group (van den Bogert et al., 2013). Regarding *Selenomonas*, prior FMT studies have shown its therapeutic potential through glucose fermentation pathways, with increased abundance of this genus after transplantation correlating significantly with improved colonic motility and alleviation of neuropsychiatric comorbidities associated with constipation (Yang et al., 2023). Although *Haemophilus* has been considered an opportunistic pathogen in some studies, other studies have shown a negative correlation between *Haemophilus* and constipation, which is consistent with the findings of the present study (He et al., 2025; Yuan et al., 2023).

At the metabolic level, we observed marked disruptions, especially among lipid-derived mediators. The elevated level of oleamide, an endogenous fatty acid amide, is of particular interest. Oleamide is a known suppressor of intestinal peristalsis via activation of the cannabinoid receptor CB1 (Capasso et al., 2005). Notably, the therapeutic effect of the TCM formula MaZiRenWan on FC has been partly attributed to its ability to down-regulate oleamide levels (Huang et al., 2020). While these observations suggest a plausible link between oleamide elevation and constipation, and are consistent with previously proposed mechanisms, our current data are correlational and do not establish a direct causal role for oleamide in driving constipation in our cohort. This finding should therefore be interpreted as hypothesis-generating, warranting validation through targeted metabolomics and functional assays.

Beyond individual metabolites, our study suggests a potential role for sphingolipid metabolism in childhood FC. Sphingolipids, including precursors like phytosphingosine and dihydrosphingosine, are bioactive molecules known to regulate smooth muscle contraction and cell signaling (Maceyka et al., 2012; Watterson et al., 2005). The key sphingolipid mediator, sphingosine-1-phosphate (S1P), is a potent inducer of intestinal smooth muscle contraction via the interstitial cells of Cajal (ICC) (Dragusin et al., 2006; Kim et al., 2013). Disruption of S1P signaling has been reported to impair ICC function and colonic motility (Mostafa et al., 2010). In our data, the altered levels of sphingolipid pathway metabolites observed in the YINDC group are associated with reduced gastrointestinal motility. This association is consistent with previous experimental studies in which supplementation of related sphingolipids (e.g., phytosphingosine, dihydrosphingosine) promoted gastrointestinal motility. However, given the cross-sectional and correlational nature of our multi-omics approach, these associations do not imply causation. We therefore emphasize that the observed sphingolipid signatures are exploratory and hypothesis-generating, rather than definitive evidence of a direct pathogenic role. Definitive conclusions regarding their involvement in yin-deficiency constitution/FC will require targeted lipidomics, functional assays, animal models, or longitudinal/intervention studies to establish causality and dissect the underlying mechanisms.

This study used the 5 symptoms of Rome IV to diagnose FC. However, two of the symptoms showed no significant difference between the YINDC and BC group in Table 2. Based on our analysis, there might be the following two reasons: First, Rome IV diagnosis is syndromic - it requires a combination of > 2 symptoms, not any single one. Although “excessive stool retention” and “rectal fecal mass” showed no significant differences between groups, the YINDC group still met the diagnostic threshold through the other three symptoms, all of which were significantly more prevalent than in the BC group. Second, different symptoms have different clinical sensitivity: “excessive stool retention” and “rectal fecal mass” are generally observed in more severe or prolonged constipation, and their absence in some YINDC cases does not negate the diagnosis. Therefore, the lack of significant differences in these two items does not compromise the validity of group classification.

Several limitations of this study should be acknowledged. The current two-group design cannot separate the independent effects of yin-deficiency constitution and functional constipation. The characteristics of the yin-deficiency constitution and functional constipation are defined in contrast to individuals with a balanced constitution who do not suffer from constipation. An infant-specific stool scale might provide greater accuracy; future studies should consider using such validated infant-specific tools. The sample size, while informative, is relatively modest, potentially limiting generalizability; future larger multi-center cohorts are needed. This study may have missing variables (e.g., the interval between feeding and sampling, medication history that falls outside the exclusion criteria, environmental exposures, detailed dietary intake, laxative use, and hydration status). The use of 16S rRNA sequencing restricts taxonomic resolution to the genus level and precludes functional inference; subsequent shotgun metagenomics or metatranscriptomics could provide species-level identification and functional insights. The current differential taxa are exploratory candidate features, and covariate-adjusted methods such as ANCOM-BC or MaAsLin2 are needed in future validation studies. Non-targeted metabolomics may carry a risk of false positives for metabolites. Signals for urinary metabolites have not yet been standardized, which may affect the comparability of metabolite intensities across individuals; therefore, the relevant research findings should be interpreted cautiously. Lastly, the correlational nature of this study requires mechanistic validation. Future FMT experiments, targeted metabolite manipulation (e.g., oleamide, S1P), and intervention studies are needed to establish causality and explore therapeutic potential.

In conclusion, our integrative analysis demonstrates that infants with YINDC harbor distinct gut microbiome and metabolome profiles. The convergence of specific pro-constipation bacteria such as *Collinsella* and *Phascolarctobacterium*, a reduction in beneficial taxa including *Veillonella* and *Selenomonas*, and an imbalance in motility-regulating metabolites such as oleamide and sphingolipids, provides a novel, multi-faceted understanding of FC in this TCM constitution type.

## 5 Conclusion

This research provides a preliminary systematic characterization of gut microbial and metabolomic profiles in yin-deficiency constitution/FC infants compared with balanced non-constipated controls. We identified differential microbial taxa and metabolites affecting sphingolipid-related pathways, but these observed differences reflect the combined phenotype and cannot be disentangled to individual factors. The results offer preliminary multi-omics evidence and generate hypotheses for future mechanistic and validation studies. While they may inform targeted therapies, clinical applicability awaits replication in larger, independent populations.

## Data Availability

The datasets presented in this study can be found in online repositories. The names of the repository/repositories and accession number(s) can be found in the article/Supplementary material.
