# Supplementary material for: Gut microbiome and urinary metabolomic alterations in Chinese infants with functional constipation and yin-deficiency constitution
Source: Front Microbiol. 2026 Aug 14;17:1851053. doi: 10.3389/fmicb.2026.1851053 (PMC13522173; doi:10.3389/fmicb.2026.1851053)
Supplement: Supplementary file 1 [file Table_1.docx]

**Supplementary file 1.**

Wang Qi's Nine Body Types in Constitutional Medicine Questionnaire (0-3 Years Version).

| Experience/condition in the past year | No | Slightly | Sometimes | Often | All the  time |
| --- | --- | --- | --- | --- | --- |
| (1) Was your child energetic? | 1 | 2 | 3 | 4 | 5 |
| (2) Did your child cry/talk loudly? | 1 | 2 | 3 | 4 | 5 |
| (3) Did your child have a quick response? | 1 | 2 | 3 | 4 | 5 |
| (4) Did your child’s complexion look yellowish, not as rosy as other children? | 1 | 2 | 3 | 4 | 5 |
| (5) Did your child catch colds more easily than other children? | 1 | 2 | 3 | 4 | 5 |
| (6) Was your child always quiet? | 1 | 2 | 3 | 4 | 5 |
| (7) Did your child talk (or cry) feebly? | 1 | 2 | 3 | 4 | 5 |
| (8) Did your child refuse to eat or eat less than other children? | 1 | 2 | 3 | 4 | 5 |
| (9) Did your child sweat more easily than other children during activities? | 1 | 2 | 3 | 4 | 5 |
| (10) Did your child always have cold hands and feet? | 1 | 2 | 3 | 4 | 5 |
| (11) Did your child get diarrhea easily if he or she got cold or ate something cold (like fruit)? | 1 | 2 | 3 | 4 | 5 |
| (12) Did your child urinate a lot at night (or need to change diaper at night)? | 1 | 2 | 3 | 4 | 5 |
| (13) Did your child look pale? | 1 | 2 | 3 | 4 | 5 |
| (14) Did the palms of your child’s hands or the soles of your child’s feet feel hot? | 1 | 2 | 3 | 4 | 5 |
| (15) Did your child's skin and lips often dry? | 1 | 2 | 3 | 4 | 5 |
| (16) Was your child thin than usual? | 1 | 2 | 3 | 4 | 5 |
| (17) Was your child always excited? | 1 | 2 | 3 | 4 | 5 |
| (18) Did your child often perspire when sleeping? | 1 | 2 | 3 | 4 | 5 |
| (19) Did your child often have dry stools? | 1 | 2 | 3 | 4 | 5 |
| (20) Was your child's tongue coating unevenly distributed, like the map (as shown on the right)?  | 1 | 2 | 3 | 4 | 5 |
| (21) Was your child overweight? | 1 | 2 | 3 | 4 | 5 |
| (22) Did your child not like activities? | 1 | 2 | 3 | 4 | 5 |
| (23) Did your child have an abundance of phlegm when he or she caught colds? | 1 | 2 | 3 | 4 | 5 |
| (24) Did your child’s tongue have a thick coating (like the picture on the right)?  | 1 | 2 | 3 | 4 | 5 |
| (25) Was your child prone to red eyes or eye excrement? | 1 | 2 | 3 | 4 | 5 |
| (26) Did your child get eczema easily? | 1 | 2 | 3 | 4 | 5 |
| (27) Did your child often have yellow and smelly urine? | 1 | 2 | 3 | 4 | 5 |
| (28) Did your child sweat easily? | 1 | 2 | 3 | 4 | 5 |
| (29) Did your child's stool stick to the bedpan and can't be flush easily (or clean easily)? | 1 | 2 | 3 | 4 | 5 |
| (30) Did your child usually look gloomy？ | 1 | 2 | 3 | 4 | 5 |
| (31) Did black or purple bruises appear on your child's skin for no reason? | 1 | 2 | 3 | 4 | 5 |
| (32) Did your child's skin of the body or the skin on the neck, armpit or thigh root become darker than usual? | 1 | 2 | 3 | 4 | 5 |
| (33) Were your child’s lips darker, more blue than usual? | 1 | 2 | 3 | 4 | 5 |
| (34) Was your child shy about going to a new place or meeting new people for the first time? (stick close to mom or hide behind) | 1 | 2 | 3 | 4 | 5 |
| (35) Did your child stay upset and squirmy for days on end when he or she was in a bad mood? | 1 | 2 | 3 | 4 | 5 |
| (36) Was your child still squeamish after meeting a stranger for 15 minutes？ | 1 | 2 | 3 | 4 | 5 |
| (37) Did your child get scared or frightened easily？ | 1 | 2 | 3 | 4 | 5 |
| (38) Did your child sneeze easily? | 1 | 2 | 3 | 4 | 5 |
| (39) Was your child prone to nasal congestion and runny nose? | 1 | 2 | 3 | 4 | 5 |
| (40) Was your child’s skin usually itchy? | 1 | 2 | 3 | 4 | 5 |
| (41) Did your child cough and wheeze when the seasons changed, the temperature changed or he or she smelled something strange? | 1 | 2 | 3 | 4 | 5 |
| (42) Did your child ever have any allergies? | 1 | 2 | 3 | 4 | 5 |
| (43) Did your child sleep well? | 1 | 2 | 3 | 4 | 5 |

These items were scored on a 5-point Likert scale, with higher scores indicating a higher likelihood of having the given constitution. The scores for each constitution were calculated as follows: raw score = the sum of the scores of each item, conversion score = [(raw score -number of items)/(number of items × 4)] × 100. To ensure the reliability of the results, BC individuals were considered as those with ≥60 points on the BC conversion score, while unbalanced constitution conversion scores were defined as <30 points; YINDC individuals were considered as those with ≥40 on the YINDC conversion score.
