# Supplementary material for: Gut microbiome and urinary metabolomic alterations in Chinese infants with functional constipation and yin-deficiency constitution
Source: Front Microbiol. 2026 Aug 14;17:1851053. doi: 10.3389/fmicb.2026.1851053 (PMC13522173; doi:10.3389/fmicb.2026.1851053)
Supplement: Supplementary file 2 [file Table_2.docx]

**Supplementary file 2.**

Influencing factors of the YINDC and BC groups

| **V****ariables** | | **YINDC**  **（*n* = 31）** | **BC**  **（*n* = 31）** | ***P-*value** |
| --- | --- | --- | --- | --- |
| Age (months) |  | 21.84 ± 5.91 | 19.06 ± 4.88 | 0.439 |
| Gender | Boys | 13 | 17 | 0.309 |
|  | Girls | 18 | 14 |  |
| Delivery Mode | Vaginal Delivery | 18 | 17 | 0.798 |
|  | Caesarean Delivery | 13 | 14 |  |
| Feeding Pattern | Breast Feeding | 18 | 20 | 0.602 |
|  | Non-Breast Feeding | 13 | 11 |  |
