# Supplementary material for: Gut microbiome and urinary metabolomic alterations in Chinese infants with functional constipation and yin-deficiency constitution
Source: Front Microbiol. 2026 Aug 14;17:1851053. doi: 10.3389/fmicb.2026.1851053 (PMC13522173; doi:10.3389/fmicb.2026.1851053)
Supplement: Supplementary file 4 [file Table_4.docx]

**Supplementary File 4.**

Number of reads retained per sample

| **Sample ID** | **Input** |
| --- | --- |
| BC1 | 122252 |
| BC2 | 125683 |
| BC3 | 120907 |
| BC4 | 125511 |
| BC5 | 125295 |
| BC6 | 123810 |
| BC7 | 131097 |
| BC8 | 116280 |
| BC9 | 124936 |
| BC10 | 130113 |
| BC11 | 116811 |
| BC12 | 96941 |
| BC13 | 128662 |
| BC14 | 120385 |
| BC15 | 133850 |
| BC16 | 128747 |
| BC17 | 118887 |
| BC18 | 122982 |
| BC19 | 120598 |
| BC20 | 115661 |
| BC21 | 126654 |
| BC22 | 111522 |
| BC23 | 120365 |
| BC24 | 110149 |
| BC25 | 96979 |
| BC26 | 105779 |
| BC27 | 106886 |
| BC28 | 88961 |
| BC29 | 111542 |
| BC30 | 102599 |
| BC31 | 91456 |
| YINDC1 | 106825 |
| YINDC2 | 109332 |
| YINDC3 | 90731 |
| YINDC4 | 101218 |
| YINDC5 | 98879 |
| YINDC6 | 108106 |
| YINDC7 | 119848 |
| YINDC8 | 110993 |
| YINDC9 | 106951 |
| YINDC10 | 110923 |
| YINDC11 | 98839 |
| YINDC12 | 109141 |
| YINDC13 | 109235 |
| YINDC14 | 119817 |
| YINDC15 | 108751 |
| YINDC16 | 108530 |
| YINDC17 | 86394 |
| YINDC18 | 96382 |
| YINDC19 | 93371 |
| YINDC20 | 103641 |
| YINDC21 | 96479 |
| YINDC22 | 88740 |
| YINDC23 | 101776 |
| YINDC24 | 97833 |
| YINDC25 | 88782 |
| YINDC26 | 103682 |
| YINDC27 | 95987 |
| YINDC28 | 102930 |
| YINDC29 | 107027 |
| YINDC30 | 83294 |
| YINDC31 | 94818 |
