# Supplementary material for: Gut microbiome and urinary metabolomic alterations in Chinese infants with functional constipation and yin-deficiency constitution
Source: Front Microbiol. 2026 Aug 14;17:1851053. doi: 10.3389/fmicb.2026.1851053 (PMC13522173; doi:10.3389/fmicb.2026.1851053)
Supplement: Supplementary file 5 [file Table_5.docx]

| **A** | **B** |
| --- | --- |
|  |  |
| **C** | **D** |
|  |  |

**Supplementary file 5.** The total ion flow plots of the QC samples in positive modes (A,B) and negative modes (C,D).
