# Supplementary material for: Gut microbiome and urinary metabolomic alterations in Chinese infants with functional constipation and yin-deficiency constitution
Source: Front Microbiol. 2026 Aug 14;17:1851053. doi: 10.3389/fmicb.2026.1851053 (PMC13522173; doi:10.3389/fmicb.2026.1851053)
Supplement: Supplementary file 6 [file Table_6.docx]

**S**upplemental file **6.**

Differential urine metabolites between the YINDC and BC groups

| **Name** | **RT (s)** | **m/z** | **Adduction** | **VIP** | ***P-***  **value** | **Score** | **FDR-adjusted *P*- value** | **Fold change** | **Identification level** | **Database source** |
| --- | --- | --- | --- | --- | --- | --- | --- | --- | --- | --- |
| (r)-(+)-arachidonyl-1'-hydroxy-2'-propylamide | 68.39 | 362.33 | [M+H]+ | 1.9 | 5.17649E-05 | 0.97 | 0.0042 | 1.6326 | 2 | Metlin |
| 2,4,6-tri-tert-butylaniline | 69.67 | 246.24 | [M+H-CH4]+ | 5.0 | 1.65184E-05 | 0.97 | 0.0016 | 1.9509 | 2 | Metlin |
| 3,6,9,12-tetraoxatetracosan-1-ol | 33.10 | 363.31 | [M+H]+ | 2.8 | 1.71167E-05 | 0.77 | 0.0016 | 1.2263 | 2 | Metlin |
| Dodecanoic acid, 12-[[(cyclohexylamino)carbonyl]amino]- | 33.97 | 198.19 | [M+H-C7H13NO2]+ | 3.0 | 5.06916E-08 | 0.90 | 0.0000 | 0.5880 | 2 | Metlin |
| Erucamide | 33.62 | 338.34 | [M+H]+ | 8.5 | 4.78233E-06 | 0.99 | 0.0006 | 0.5253 | 1 | In-house database |
| Linoleoylglycine | 34.56 | 263.24 | [M+H-C2H5O2N]+ | 6.4 | 9.35279E-15 | 0.96 | 0.0000 | 0.5829 | 2 | Metlin |
| N-lauroyl-d-erythro-sphinganine | 32.63 | 484.47 | [M+H]+ | 2.3 | 7.05535E-06 | 0.90 | 0.0008 | 1.9075 | 2 | Metlin |
| N-myristoylsphinganine | 32.87 | 512.50 | [M+H]+ | 4.1 | 6.05758E-05 | 0.86 | 0.0048 | 1.5188 | 2 | Metlin |
| N-octanoylsphingosine | 65.05 | 390.36 | [M+H-2H2O]+ | 1.8 | 5.9595E-08 | 0.95 | 0.0000 | 21.0678 | 2 | Metlin |
| Oleamide | 34.06 | 282.28 | [M+H]+ | 30.5 | 3.29051E-16 | 0.98 | 0.0000 | 0.4736 | 2 | Metlin |
| Palmitamide | 34.29 | 256.26 | [M+H]+ | 9.5 | 6.88426E-19 | 1.00 | 0.0000 | 0.4649 | 2 | Metlin |
| Phytosphingosine | 66.81 | 318.30 | [M+H]+ | 5.0 | 1.14922E-05 | 0.98 | 0.0012 | 1.6279 | 1 | In-house database |
| Pro-Trp | 62.29 | 302.31 | [M+H]+ | 5.4 | 2.86363E-05 | 1.00 | 0.0025 | 1.4433 | 1 | In-house database |
| Sphingosine | 101.49 | 282.28 | [M+H-H2O]+ | 1.3 | 0.000775914 | 0.97 | 0.0408 | 0.5876 | 1 | In-house database |
| Zerumbone | 35.98 | 119.09 | [M+H-C6H12O]+ | 5.1 | 8.76379E-07 | 0.99 | 0.0001 | 0.8533 | 2 | Metlin |
