# Supplementary material for: Gut microbiome and urinary metabolomic alterations in Chinese infants with functional constipation and yin-deficiency constitution
Source: Front Microbiol. 2026 Aug 14;17:1851053. doi: 10.3389/fmicb.2026.1851053 (PMC13522173; doi:10.3389/fmicb.2026.1851053)
Supplement: Supplementary file 7 [file Table_7.docx]

|  |  |
| --- | --- |
| **A** (r)-(+)-arachidonyl-1'-hydroxy-2'-propylamide | **B** 2,4,6-tri-tert-butylaniline |
|  |  |
| **C** 3,6,9,12-tetraoxatetracosan-1-ol | **D** Dodecanoic acid, 12-[[(cyclohexylamino)carbonyl]amino]- |
|  |  |
| **E** Erucamide | **F** Linoleoylglycine |
|  |  |
| **G** N-lauroyl-d-erythro-sphinganine | **H** Oleamide |
|  |  |
| **I** N-myristoylsphinganine | **J** Palmitamide |
|  |  |
| **K** N-octanoylsphingosine | **L** Sphingosine |
|  |  |
| **M** Phytosphingosine | **N** Zerumbone |
|  |  |
| **O** Pro-Trp |  |

**Supplementary File 7**

MS/MS spectra of differential metabolites.
